# Genetic evidence for differential selection of grain and embryo weight during wheat evolution under domestication

**DOI:** 10.1093/jxb/erv249

**Published:** 2015-05-27

**Authors:** Guy Golan, Adi Oksenberg, Zvi Peleg

**Affiliations:** The Robert H. Smith Institute of Plant Sciences and Genetics in Agriculture, The Hebrew University of Jerusalem, Rehovot 7610001, Israel

**Keywords:** Early vigour, embryo, embryo/endosperm ratio, endosperm, grain size, tetraploid wheat, *Triticum turgidum* ssp. *dicoccoides*, wheat domestication.

## Abstract

Wheat is one of the Neolithic founder crops domesticated ~10 500 years ago. Following the domestication episode, its evolution under domestication has resulted in various genetic modifications. Grain weight, embryo weight, and the interaction between those factors were examined among domesticated durum wheat and its direct progenitor, wild emmer wheat. Experimental data show that grain weight has increased over the course of wheat evolution without any parallel change in embryo weight, resulting in a significantly reduced (30%) embryo weight/grain weight ratio in domesticated wheat. The genetic factors associated with these modifications were further investigated using a population of recombinant inbred substitution lines that segregated for chromosome 2A. A cluster of loci affecting grain weight and shape was identified on the long arm of chromosome 2AL. Interestingly, a novel locus controlling embryo weight was mapped on chromosome 2AS, on which the wild emmer allele promotes heavier embryos and greater seedling vigour. To the best of our knowledge, this is the first report of a QTL for embryo weight in wheat. The results suggest a differential selection of grain and embryo weight during the evolution of domesticated wheat. It is argued that conscious selection by early farmers favouring larger grains and smaller embryos appears to have resulted in a significant change in endosperm weight/embryo weight ratio in the domesticated wheat. Exposing the genetic factors associated with endosperm and embryo size improves our understanding of the evolutionary dynamics of wheat under domestication and is likely to be useful for future wheat-breeding efforts.

## Highlight

Genetic and phenotypic analysis of wild and domesticated tetraploid wheat suggests differential selection of grain and embryo weight during wheat evolution under domestication.

## Introduction

Wheat (*Triticum* spp.) is one of the Neolithic founder crops, domesticated alongside other cereals and legumes in the Near-Eastern Fertile Crescent ~10 500 years ago (Lev-Yadun *et al.*, 2000). Today, wheat occupies 215 million hectares (16% of all cropland) with ~700 million tonnes produced annually, providing about one-fifth of the calories and protein consumed by humans (http://faostat.fao.org). The domestication and (subsequent) evolution under domestication of wheat has occurred through a number of genetic changes, referred to as the ‘domestication syndrome’ (Hammer, 1984), which have affected a variety of morpho-physiological traits (Peleg *et al.*, 2011; Abbo *et al.*, 2012, 2014b).

Wheat grain (caryopsis), a single-seeded fruit, has been a major target for selection since the dawn of agriculture (Harlan, 1992). Grain weight is a complex quantitative trait under polygenic control which is influenced by various genetic interactions at all stages of growth. Grain weight is positively associated with agronomic yield and is a very stable yield component, with relatively high heritability. Quantitative trait loci (QTL) affecting grain weight, grain size, and grain shape have been reported on most wheat chromosomes (Börner *et al.*, 2002; Huang *et al.*, 2003; Breseghello and Sorrells, 2006, 2007; Kumar *et al.*, 2006; Gegas *et al.*, 2010; Peleg *et al.*, 2011; Simmonds *et al.*, 2014). Phenotypic variation in grain weight and size is also attributed to environmental factors such as water availability and extreme temperatures, which affect the rate and duration of the grain-filling process.

The grain of domesticated durum wheat [*T. turgidum* ssp. *durum* (Desf.) MacKey] is composed of pericarp and testa (~12%), embryo (~1.6%), and a prominent and persistent endosperm that accounts for ~86% of the grain weight (Kulp and Ponte, 2000). Endosperm development is known to be under strong maternal control (Felker *et al.*, 1985). The floral cavity, which is determined by the size and shape of the lemma and the palea at anthesis, is thought to physically restrict grain volume (Millet, 1986). From anthesis to physiological maturity, the rate and, to a lesser extent, the duration of grain-filling determine the final endosperm size (Egli, 2006). The endosperm nourishes the embryo during seed development and germination. A failure in endosperm development will halt the growth of the developing embryo, underscoring the embryo’s dependency on the endosperm (Lafon-Placette and Köhler, 2014). A strong positive correlation between embryo weight and seed weight has been reported in monocot (Bremner *et al.*, 1963; López-Castañeda *et al.*, 1996; Aparicio *et al.*, 2002; Richards and Lukacs, 2002) and eudicot (Hedley and Ambrose, 1981) species. While the proportion of embryo to endosperm varies widely among species, it is relatively uniform within individual species (Nagasawa *et al.*, 2013).

It is well accepted that larger grains promote early vigour in cereal crops (Bremner *et al.*, 1963; Evans and Bhatt, 1977; Lafond and Baker, 1986; Revilla *et al.*, 1999; Aparicio *et al.*, 2002). Similarly, previous studies indicated that embryo size is associated with seedling vigour. Based on the close correlation between seedling growth and embryo size, Ashby (1930, 1932) suggested that the hybrid vigour of maize (*Zea mays* L.) is due to the maintenance of an initial advantage in embryo size rather than grain size. Furthermore, in different cereal species with similarly sized grain, embryo weight has been found to account for most of the variation in seedling vigour (López-Castañeda *et al.*, 1996). By contrast, Bremner *et al.* (1963) demonstrated that endosperm weight affects early vigour, while embryo weight makes only a negligible contribution to the phenotype.

Archaeobotanical evidence from the Fertile Crescent region suggests that there was an increase in grain size following the domestication of allo-tetraploid (2*n*=4*x*=28; genome BBAA) wild emmer wheat [*T. turgidum* ssp. *dicoccoides* (Körn.) Thell.] and the subsequent evolution of the domesticated form (*T. turgidum* ssp. *dicoccum* Schrank) (Willcox, 2004; Fuller, 2007; Fuller *et al.*, 2012). However, to the best of our knowledge, there have been no similar reports regarding embryo weight. In the current study the aim was to (i) examine the modifications in grain weight and embryo weight along wheat evolution, (ii) investigate the genetic basis of embryo weight, grain weight, and grain shape on chromosome 2A, and (iii) investigate the relationships between embryo weight and early seedling vigour. Our results shed new light on the changes in endosperm to embryo ratio during wheat evolution under domestication and the selection forces driving these changes are discussed.

## Materials and methods

### Plant material and growing conditions

A panel of tetraploid wheat genotypes comprising 12 wild emmer accessions and 12 domesticated durum wheat cultivars (see Supplementary Table S1 at *JXB* online) was grown in an insect-proof screen-house at the experimental farm of the Hebrew University of Jerusalem in Rehovot, Israel (34°47′ N, 31°54′ E; 54 m above sea level). The soil at this location is a brown-red, degrading sandy loam (Rhodoxeralf) composed of 76% sand, 8% silt, and 16% clay. A completely random block design was used. The trial was replicated three times with 75-cm-long plots, each containing five plants. Plants were treated with pesticides to protect against pathogens and insect pests and the plots were weeded by hand once a week.

A population of 94 homozygous recombinant inbred substitution lines (RISL) derived from a cross between the durum wheat cultivar ‘Langdon’ (LDN) and the substitution line DIC-2A (Joppa, 1993) was used for grain and embryo characterization. The substitution line DIC-2A contained the 2A chromosome from the wild emmer accession Israel-A (FA-15-3) against the genetic background of LDN. The RISLs and their parental lines were grown in an insect-proof screen-house in a completely random arrangement with three plants for each line. The RISL population was selected based on a preliminary examination of grain and embryo size between the two parental lines. This population is a useful tool for dissecting the genetic relationship between these traits on chromosome 2A, in an otherwise homozygous LDN background. Moreover, it provides an excellent base for fine mapping and positional cloning of the genes underlying QTL.

## Phenotypic measurements

Each plot/plant was harvested, threshed, oven-dried (35 °C for 96h) and weighed. Grains were counted using a seed counter (DATA Count S-25, DATA TECHNOLOGIES, Jerusalem, Israel) and weighed (GW=grain weight). Embryos were dissected from mature dry grains using forceps. Five replicates (each with three embryos) per plant were weighed on a microbalance (M2P, Sartorius, Göttingen, Germany) to obtain embryo-weight (EmW) data. Grains were scanned using a flatbed scanner and grain length (GL) and width (Gwid) were determined using the GrainScan software (Whan *et al.*, 2014). The lengths and widths of the embryos of the parental lines were documented using a binocular microscope (SZX16, Olympus, Tokyo, Japan) equipped with a DP73 digital camera and analysed using Image J software (http://imagej.nih.gov/ij).

Nine RISLs carrying different grain weight (GW) and embryo weight (EmW) alleles (see Supplementary Table S5 at *JXB* online), as well as their two parental lines (LDN and DIC-2A), were characterized for early seedling vigour and the effect of their allelic combination was tested. Only seeds weighing 55–60mg were selected for this experiment. Six uniform seeds of each genotype were germinated in Petri dishes with germination paper for 2 d. Germinated seeds were then sown (2cm depth) in plastic containers (26×16.5×11cm) filled with a mixture of fine peat, with five replicates for each genotype. Seedlings were placed in a temperature-controlled greenhouse (24/16 °C day/night) and watered daily. Fourteen days after sowing, the above-ground shoots were harvested, oven-dried (60 °C for 72h), and weighed.

### Statistical analysis of phenotypic data

The JMP^®^ ver. 11 statistical package (SAS Institute, Cary, NC, USA) was used for statistical analyses. The homogeneity of variances between wild emmer and durum wheat was examined using Bartlett’s test and differences between mean values were examined using Welch’s test. The associations among grain and embryo characteristics were studied using a Pearson correlation analysis. Differences between the three parental lines (i.e. LDN, Israel-A, and DIC-2A) were analysed using Tukey HSD at *P* ≤0.05. All phenotypic variables that were subjected to QTL analysis had been tested for normal distribution. Differences in seedling vigour were analysed using Dunnet’s test at *P* ≤0.05.

### Genetic analyses

DNA was extracted from fresh leaf tissue (~200mg) from individual 2–3-week-old plants using a standard CTAB protocol. Microsatellite marker sequences and amplification conditions were as described in the GrainGenes 2.0 database (http://wheat.pw.usda.gov). The primers developed in the current study (*Xhuj*001–004) are listed in Supplementary Table S2 at *JXB* online. The *Xhuj* primers were developed based on sequences deposited in the IWGSC database (http://www.wheatgenome.org), corresponding to sequences used in the 90K iSelect assay (Avni *et al.*, 2014; Wang *et al.*, 2014). For markers *Xhuj*001–003, PCR conditions were as follows (20 μl total volume): Taq Ready Mix (2×) (Hy Labs, Rehovot, Israel), 100ng of template DNA, and 10 pmol each of the forward and reverse primers. Amplification was carried out in a thermal cycler (T-100, BIO-RAD, Hercules, CA, USA) running the following programme: 5min at 94 °C; 34 cycles of 30 s at 94 °C, 30 s at 60 °C, 30 s (90 s for *Xhuj*004) at 72 °C with a final extension of 72 °C for 7min. For *Xhuj*003, following amplification, products were digested with Fnu4HI (New England Bio Labs, Ipswich, MA, USA) in the PCR reaction tube according to the manufacturer’s instructions. PCR products were visualized using 2–5% agarose gels stained with ethidium bromide. In total, 30 markers were used to genotype the 94 RISLs.

For each segregating marker, a χ^2^ analysis was performed to test for deviation from the 1:1 expected segregation ratio. Linkage analyses and map construction were performed based on the evolutionary strategy algorithm included in the MultiPoint package (http://www.MultiQTL.com), as previously described by Mester *et al*. (2003a, b, 2004) and Peleg *et al.* (2008).

QTL analysis was performed with the MultiQTL package, using the general interval mapping for a RIL-selfing population as described by Peleg *et al.* (2011). To examine the possibility of two linked QTLs, the hypotheses that a single locus or two linked loci on the considered chromosome affect(s) one or two quantitative traits were tested by running 5 000 permutation tests (Churchill and Doerge, 1994). The hypothesis that one locus on the chromosome has an effect on a given trait (H_1_) was compared with the null hypothesis (H_0_) that the locus had no effect on that trait. Once the genetic model was chosen, 5 000 bootstrap samples were run to estimate the standard deviation of the main parameters: locus effect, its chromosomal position, its LOD score and the proportion of explained variation (PEV).

## Results

### Grain weight and embryo weight among wild and domesticated wheat

**Fig. 1.**
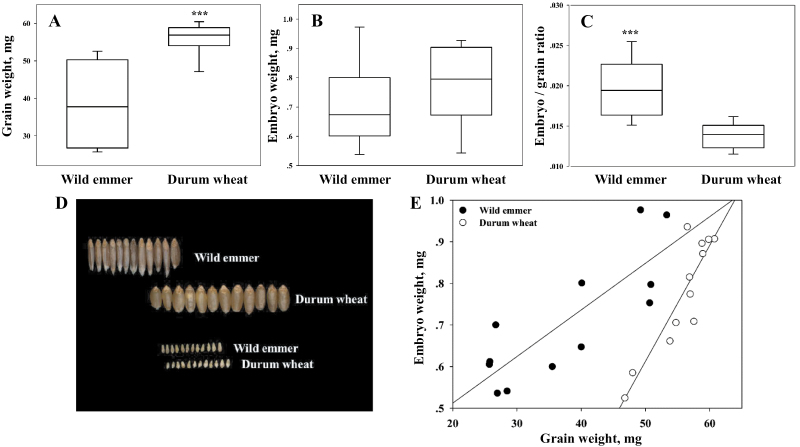
A comparison of grain and embryo weights between wild emmer wheat and durum wheat. Boxplot of (A) grain weight, (B) embryo weight, and (C) embryo weight/grain weight ratio among 12 wild emmer accessions and 12 cultivars of durum wheat. (D) A representative photo demonstrating the variation in grain and embryo size. Grains and embryos were arranged in order of their grain weight, from left to right. (E) Correlation analysis between grain weight and embryo weight. *** indicates a significant difference (*P* <0.0001). This figure is available in colour at *JXB* online.)

The grain and embryo weights of a set of 12 wild emmer wheat accessions and 12 durum wheat cultivars were analysed (Fig. 1). An analysis of variance showed a significant difference in grain weight between the two sub-species (37.7 versus 55.7mg, respectively; Fig. 1A), with an overlap between wild emmer (range 25.7–53.3mg) and the durum wheat (range 46.7–60.7mg; Fig. 1A, D). The coefficient of variance (CV) among the wild emmer accessions (29.23) was higher than that observed among the durum cultivars (7.87). Embryo weight did not differ between the studied wild emmer and durum lines (0.71 versus 0.77mg, respectively; Fig. 1B, D), with minor differences in CV (21.14 and 17.71, respectively). Among the wild emmer germplasm, a significantly higher embryo weight/grain weight ratio (0.020 versus 0.014, *P* <0.0001; Fig. 1C) was documented. A significant correlation between embryo weight and grain weight was found among both the wild emmer wheat accessions and the durum cultivars (*r*=0.82, *P* <0.001 and *r*=0.89, *P* <0.0001, respectively; Fig. 1E).

### Phenotypic characterization of the RISL population

**Fig. 2.**
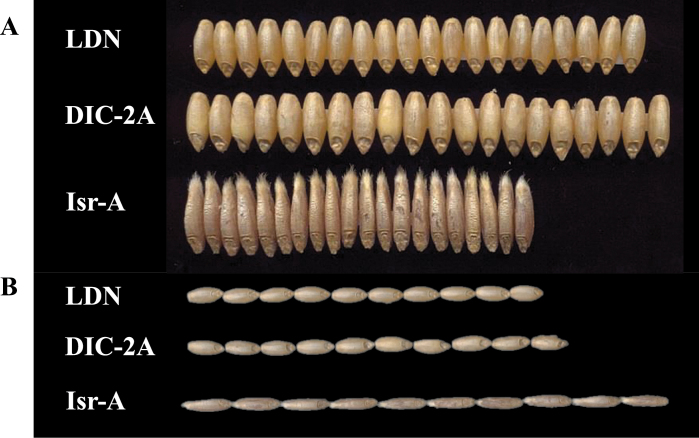
Grain characteristics of the durum wheat parental line Langdon (LDN), the substitution line DIC-2A, and the wild emmer chromosome donor Israel-A (Isr-A). (A) Representative photo showing grain width (*n*=20) and (B) grain length (*n*=10). This figure is available in colour at *JXB* online.)

**Fig. 3.**
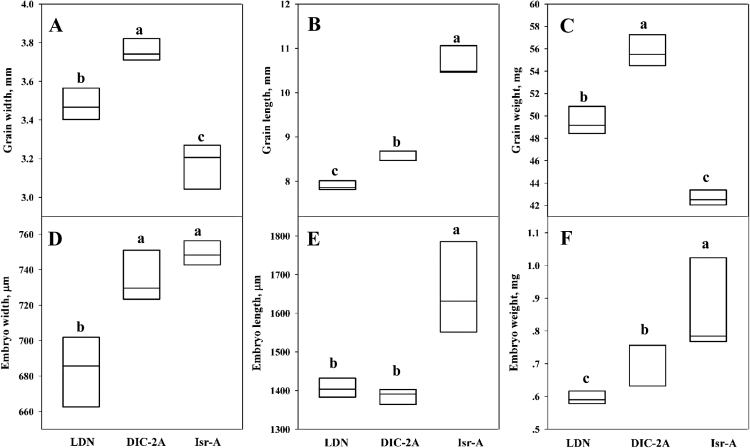
Boxplot of the grain weight, embryo weight, and shape of the two parental lines, Langdon (LDN) and DIC-2A, and the wild emmer chromosome donor, Israel-A (Isr-A). (A) Grain width, (B) grain length, (C) grain weight, (D) embryo width, (E) embryo length, and (F) embryo weight. Values are means ±SD (*n*=3). Different letters indicate significant differences, as determined by Tukey LSD test (*P* ≤0.05).

The substitution line DIC-2A had larger grains than the domesticated parental line (LDN); whereas Isr-A was characterized by long, narrow grain (Fig. 2). A detailed analysis of grain characteristics showed that DIC-2A had significantly wider grains (3.75mm) than LDN and Isr-A (3.47 and 3.17mm, respectively; Figs 2A, 3A). Isr-A had the longest grain (10.66mm; Fig. 2B); whereas DIC-2A and LDN had significantly shorter grain (8.61 and 7.89mm, respectively; Figs 2B, 3B). DIC-2A had heavier grain than LDN (55.8 versus 49.6mg, respectively); whereas the Isr-A grain was significantly lighter than that of both of the other lines (42.03mg; Fig. 3C).

Embryo width did not differ between Isr-A and DIC-2A (740 and 730 μm, respectively); whereas LDN had significantly narrower embryos (Fig. 3D). Isr-A had significantly longer embryos (1656 μm) than the other lines, but there was no difference in embryo length between LDN and DIC-2A (Fig. 3E). The DIC-2A embryos were significantly heavier than those of LDN (0.72 versus 0.59mg, respectively). Isr-A had the heaviest embryos (0.9mg; Fig. 3F).

**Fig. 4.**
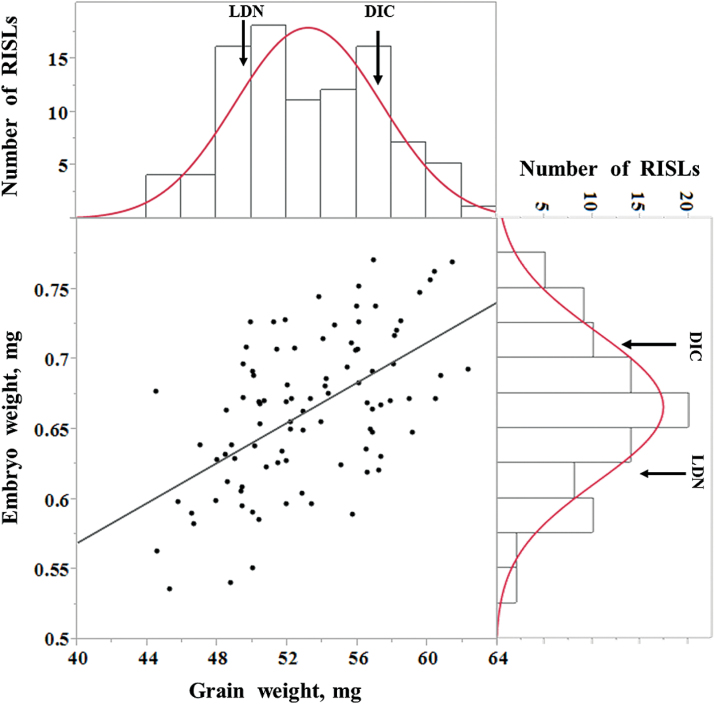
Correlation (*r*) between grain weight and embryo weight and frequency distribution of the traits among the 94 recombinant inbred substitution lines of Langdon×DIC-2A. Data are means of three replicated plants. Arrows indicate the values of the parental lines Langdon (LDN) and DIC-2A (DIC).

Frequency distributions of the continuous phenotypic traits among the RISL are presented in Fig. 4 and in Supplementary Fig. S1 at *JXB* online. All variables were normally distributed, with considerable transgressive segregation. A correlation analysis of grain characteristics revealed significant positive correlation among these traits. (see Supplementary Table S3 at *JXB* online). A positive correlation was observed between grain weight and embryo weight (*r*=0.56, *P* <0.0001; Fig. 4).

### Genetic linkage map and QTL analysis

A genetic linkage map of chromosome 2A was constructed for the 94 RISLs. A skeleton map, comprising 30 markers and encompassing a genetic distance of 125 cM at an average spacing of 4.8 cM between markers, was used for QTL mapping.

**Fig. 5.**
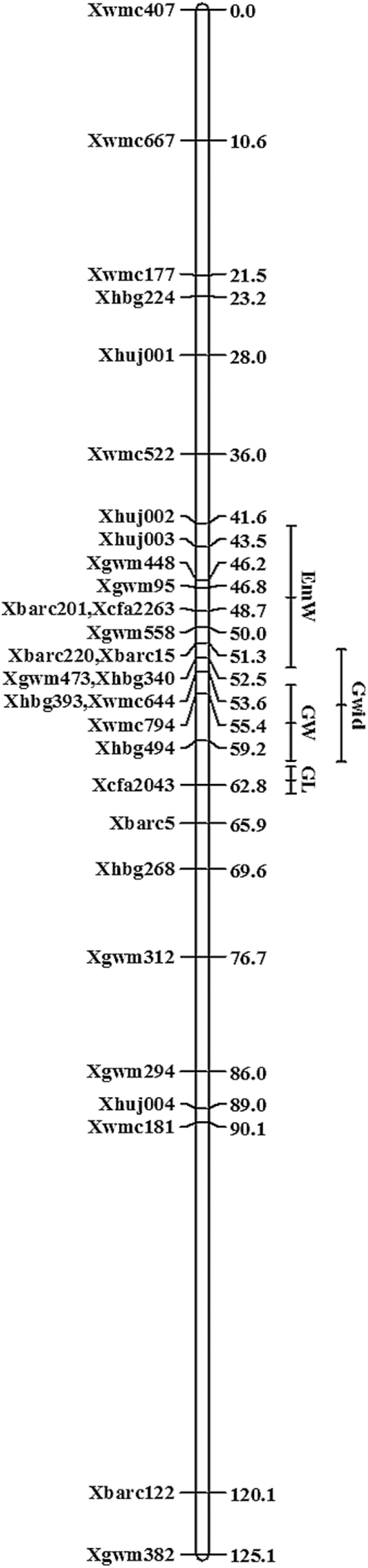
Likelihood intervals for quantitative loci (QTL) associated with grain weight (GW), embryo weight (EmW), grain length (GL), and grain width (Gwid) in recombinant inbred substitution lines of Langdon×DIC-2A.

QTL analysis revealed two distinct loci that regulate grain and embryo weight near the centromeric region of chromosome 2A. The embryo weight (EmW) QTL had a LOD score of 7.7 and was found to be located on the short arm of chromosome 2AS, explaining 32% of the variance in this trait. Higher EmW was conferred by the wild emmer allele (Fig. 5; see Supplementary Table S4 at *JXB* online). The grain weight (GW) QTL had a LOD score of 12.8 and was found to be located on the long arm of chromosome 2AL, explaining 48% of the variance in this trait. Higher GW was conferred by the wild emmer allele (Fig. 5; see Supplementary Table S4 at *JXB* online). The QTL for GW co-localized with the QTL for grain width (Gwid; LOD 9.75; Fig. 5; see Supplementary Table S4 at *JXB* online). The QTL for grain length (GL; LOD 12.4) was found to be distal to the GW locus. Longer grains were associated with the wild emmer allele and the QTL explained 45.6% of the variance in grain length (Fig. 5; see Supplementary Table S4 at *JXB* online).

**Fig. 6.**
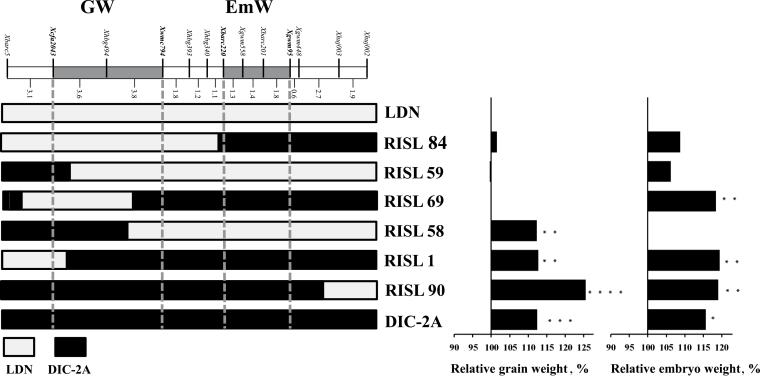
Effects of GW and EmW loci on grain weight and embryo weight. Graphical genotyping of six recombinant inbred substitution lines (RISL) and the two parental lines. Langdon (LDN) and DIC-2A. The effects of each genotype on GW and EmW are shown as the percentage difference relative to LDN. *, **, *** and **** indicates a significant difference at *P* ≤0.05, 0.01, 0.001 and 0.0001.

**Fig. 7.**
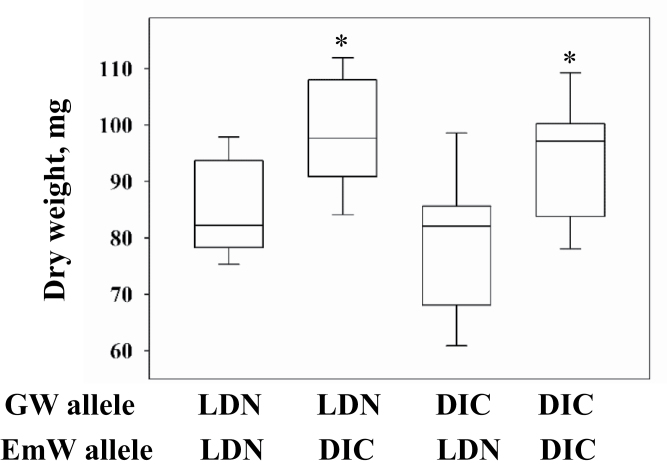
A boxplot demonstrating the effects of different grain-weight (GW) and embryo-weight (EmW) alleles on seedling weight. Domesticated and wild alleles are marked by LDN and DIC, respectively. * indicates a significant difference (*P* ≤0.05) from the group carrying both domesticated alleles.

The graphical genotyping analysis (Young and Tanksley, 1989) of six RISLs with critical recombination events in the GW and EmW region demonstrated the effects of the different alleles on grain and embryo weight and the independence of the two loci. RISLs carrying the wild allele had greater grain and embryo weights, compared with the domesticated parental line (LDN; Fig. 6). An examination of the effect of GW and EmW on early seedling vigour was carried out using similarly weighed seeds of RISL with recombination events between these two loci. The wild emmer EmW allele promoted greater seedling vigour regardless of the GW locus. The GW locus was not associated with seedling vigour (Fig. 7; see Supplementary Table S5 at *JXB* online).

## Discussion

Plant domestication and the start of an agricultural-based economy ~10 500 years ago were the most important human cultural developments. The transfer of plants from their original wild habitats to a new, human-managed environment (cultivated fields) is thought to have involved a great deal of highly conscious selection (Abbo *et al.*, 2009, 2011, 2014a). The genuine domestication-syndrome traits are the descriptors of the pristine domestication episode (Abbo *et al.*, 2014b). Subsequent, evolution of crops under domestication (Abbo *et al.*, 2012) has led to further changes in crop plants, involving numerous morpho-physiological traits that are often associated with the domestication episode (Olsen and Wendel, 2013). It should be borne in mind that changes in both genuine domestication traits (Abbo *et al.*, 2014b) and crop-evolution traits, by definition, partly involve correlated responses to selection, some of which may have been unintentional (Ladizinsky, 1998). In the current study, a genetic approach was used to investigate how the grain weight and embryo weight of wheat have changed over the course of post-domestication selection.

Archeobotanical evidence (Willcox, 2004; Fuller, 2007), as well as experimental data from the current study (Fig. 1) and other studies (Abbo *et al.*, 2014b) indicate that, in general, domesticated wheat has larger grains than its wild progenitor. The grain of domesticated wheat is usually wider and shorter, traits which are associated with heavier grain weight, while wild wheat has longer and narrower grains (Figs 1, 2). These observations are in agreement with those of a large-scale analysis of grain weight and shape carried out using a collection of ancestral wheat species which found that elite varieties with heavy grains had grains that were wider and shorter than those of wild *Triticum* species (Gegas *et al.*, 2010).

Using a set of LDN chromosome substitution lines, containing different chromosomes from the wild emmer accession Israel-A (FA-15-3), Elias *et al.* (1996) localized a grain-weight locus to chromosome 2A (DIC-2A) on which wild emmer carries the promoting allele. Here, a RISL population derived from the DIC-2A line was used to characterize this locus further and to investigate its relationship with embryo weight. Taking into consideration the polygenic nature of both traits, additional putative loci, associated with other components of grain and embryo weight, are most likely located on other wheat chromosomes. In this study, the GW locus was localized to the long arm of chromosome 2AL (Fig. 5). Similarly, a QTL for GW on chromosome 2A has been reported for several durum×wild emmer populations, with the wild allele showing higher GW values (Peng *et al.*, 2003; Peleg *et al.*, 2011; Tzarfati *et al.*, 2014). Recently, Faris *et al.* (2014) reported on a QTL affecting the number of spikelets per spike on chromosome 2AL, based on work using the same mapping population. The reduced number of spikelets per spike was conferred by the wild allele and could affect grain size. However, this QTL was mapped to a locus distal to the GW locus reported in this study.

The non-overlapping QTLs for GW and GL (Fig. 5), as well as the results of a meta-QTL analysis of several wheat mapping populations (Gegas *et al.*, 2010) suggest independent control of these two traits. By contrast, the QTL controlling grain width was co-localized with the QTL controlling grain weight. Similarly, in rice, several genes regulating grain weight have been found to regulate grain width as well, possibly by increasing cell number and cell size of the floral cavity and the endosperm (Song *et al.*, 2007; Weng *et al.*, 2008; Li *et al.*, 2011). The results of our phenotypic analyses further supported the strong association between grain weight and width.

The grain of the wild emmer chromosome donor (Isr-A) is longer, narrower, and lighter than that of the domesticated parent (LDN). Interestingly, QTLs for wider grain and higher GW were conferred by the wild emmer allele. Accordingly, DIC-2A had wider grains with significantly greater grain weight (Figs 2, 3). Taking into consideration the polygenic nature of this trait, putative decreasing alleles on other chromosomes may reduce the effect of this allele in Isr-A. Similarly, the potential benefit of re-introducing wild alleles into breeding programmes to improve various traits (e.g. resistance, quality, and yield) has previously been demonstrated in different species (reviewed by Zamir, 2001; Feuillet *et al.*, 2008).

In the present study, a novel wild locus conferring heavier embryos was mapped to a location near the GW locus. To the best of our knowledge, this is the first report of a QTL for embryo weight in wheat. The wild emmer donor (Isr-A) had significantly larger embryos than the domesticated parent (LDN; Figs 2–4). The embryo of DIC-2A was significantly wider than LDN, but no difference was observed in embryo length. Therefore, it was concluded that embryo width was the key component contributing to heavier embryo weight in DIC-2A (Fig. 3). While the embryo length of DIC-2A was similar to that of LDN, the significantly longer embryos of Isr-A suggest that embryo length is regulated by a different locus on another wheat chromosome. Remarkably, the ratio between embryo weight and grain weight of the wild parent (Isr-A) was reduced 2-fold in the domesticated parent (LDN; 2.14 versus 1.19%, respectively).

In rice, a GIANT EMBRYO (GE, encode CYP78A13, a cytochrome P450) gene has been cloned and shown to affect the endosperm/embryo ratio via regulation of cell size in the embryo and cell death in the endosperm (Nagasawa *et al.*, 2013). Similar results were obtained from a maize mutant, in which the orthologue gene encompasses an inserted transposable element (Zhang *et al.*, 2012). The GE gene was mapped to rice chromosome 7, which shares synteny with the short arm of wheat group 2 chromosomes (Somyong *et al.*, 2011). The EmW locus was mapped to chromosome 2AS, near the centromere. Further investigation is needed to determine whether the EmW locus is related to the wheat GE orthologue on 2AS.

Bremner *et al.* (1963) dissected mature wheat grains, within the same cultivar, and demonstrated that, across the grain-weight spectrum, there was a tight correlation between grain weight and embryo weight. This may suggest isometric growth of the endosperm and the embryo for at least part of the grain-development period. Similarly, a positive phenotypic correlation between grain and embryo weight was found among the wild and domesticated germplasm as well as in the RISL population (Figs 1, 4). While embryo development depends on the endosperm which can be explained by an intimate sink–source relationship, exchanges of signal molecules between the endosperm and the embryo may affect their development as well. Nagasawa *et al.* (2013) suggested that a disruption in the signalling pathway between the endosperm and the embryo altered endosperm/embryo size ratio in the GIANT EMBRYO mutant and impaired the natural correlation between endosperm and embryo size.

Regardless of the putative loci located on other wheat chromosomes, independent control of these traits was observed in the QTL analysis (Fig. 6). Considering the widespread association of seed weight and embryo weight observed in the current study, as well as in other studies (Bremner *et al.*, 1963; Hedley and Ambrose, 1981; López-Castañeda *et al.*, 1996; Aparicio *et al.*, 2002; Richards and Lukacs, 2002), one would expect to find heavier embryos in domesticated wheat, compared with wild emmer. The similarity in embryo weight between wild and domesticated wheat may suggest that the selection for larger grains targeted larger endosperm and smaller embryos. This could involve either a single pleotropic locus, as demonstrated for rice, or distinct loci, as demonstrated in the current study (Figs 5, 6).

## Concluding remarks

Genetic evidence suggests that plant domestication involved a limited number of founding genotypes (i.e. *founder effect*; Mayr, 1942), which then spread widely as the cultivated form of the plant. This phenomenon is referred as the *domestication bottleneck* (Ladizinsky, 1985). In the current study, phenotypic variation within wild and domesticated germplasm points to a genetic bottleneck associated with selection for larger grain during the evolution of wheat. On the other hand, no such pattern was found for embryo weight, as indicated by similar coefficient of variance values for the wild emmer and the durum cultivars. The increase in grain weight over the course of crop evolution could be due either to deliberate selection of desirable phenotypes by early agriculturalists (i.e. conscious selection) or to an unintended consequence of crop-cultivation processes (i.e. unconscious selection). Some researchers have suggested that larger grains may have been automatically selected due to their improved ability to emerge from deeper burial and produce vigorous seedling (Harlan *et al.*, 1973; Zohary, 2004; Purugganan and Fuller, 2009). On the other hand, the ability of wild and domesticated wheat seedlings to emerge from deep sowing (up to 7cm) was shown to be similar (Oppenheimer, 1963). Furthermore, a study of several legume crops has demonstrated that emergence from deep sowing increases with seed size in some crop species, but not others (Kluyver *et al.*, 2013). These results do not support the hypothesized automatic selection for vigorous seedlings. While it is possible that alternative, unintended selection pressures may account for differences in grain size, the possibility that early farmers may have consciously selected for larger grains cannot be ruled out.

The work presented here shows that the wild allele of EmW contributes to improved seedling vigour (Fig. 7; see Supplementary Table S5 at *JXB* online). These results and those of previous studies (Ashby, 1930, 1932; López-Castañeda *et al.*, 1996; Richards and Lukacs, 2002) suggest that embryo size is important for seedling establishment. Therefore, if, as suggested by Harlan *et al.* (1973), vigorous seedlings were automatically selected, one would expect such selection to have resulted in greater embryo weights among domesticated wheat. However, as mentioned above, grain weight has increased over the course of wheat evolution without any corresponding change in embryo weight (Fig. 1). Therefore, an alternative scenario is proposed based on conscious selection pressure favouring larger grains due to their greater number of calories, the ease of handling larger grains or other benefits considered by early farmers.

Early vigour is a key component in plants’ ability to compete with neighbouring plants (Weiner, 1990). Wild emmer grows in mixed stands with other Mediterranean grasses, such as wild barley (*Hordeum spontaneum* and *H. bulbosum*), wild oats (*Avena sterllis* and *A. barbata*) and a variety of wild legumes (Oppenheimer, 1963). Under such conditions, a seedling with greater competitive vigour is most desirable. By contrast, cultivated-field conditions are characterized by large numbers of plants of the same species. Thus, highly competitive seedlings negatively affect the growth of neighbouring plants, resulting in reduced grain yields (Milla and Morente-López, 2015). It can be hypothesized that this phenomenon, as well directed selection, may have reduced the embryo proportion of grains of domesticated wheat.

## Supplementary data

Supplementary data can be found at *JXB* online.

Supplementary Fig. S1. Frequency distribution of grain-shape parameters among the 94 RISLs.

Supplementary Table S1. List of wild emmer accessions and durum cultivars examined in this study.

Supplementary Table S2. List of primers developed for the current study.

Supplementary Table S3. Correlations between grain and embryo traits in the RISL population.

Supplementary Table S4. Biometric parameters of QTLs affecting grain and embryo parameters.

Supplementary Table. S5. The effect of EmW and GW loci on seedling vigour.

Supplementary Data
