# Supplementary material for: Genetic evidence for differential selection of grain and embryo weight during wheat evolution under domestication
Source: J Exp Bot. 2015 May 27;66(19):5703–11. doi: 10.1093/jxb/erv249 (PMC4566971; doi:10.1093/jxb/erv249)
Supplement: Supplementary Data [file supp_66_19_5703__index.html]

Genetic evidence for differential selection of grain and embryo weight during wheat evolution under domestication — Genetic evidence for differential selection of grain and embryo weight during wheat evolution under domestication — Supplementary Data 

# Genetic evidence for differential selection of grain and embryo weight during wheat evolution under domestication

## Supplementary Data

Data files

- Supplementary Data - Supplementary Data
