# Supplementary material for: Genetic evidence for differential selection of grain and embryo weight during wheat evolution under domestication
Source: J Exp Bot. 2015 May 27;66(19):5703–11. doi: 10.1093/jxb/erv249 (PMC4566971; doi:10.1093/jxb/erv249)
Supplement: Supplementary Data [file supp_erv249_jexbot146563_file001.pdf]

A Genetic Evidence for Differential Selection of Grain and Embryo Weight during Wheat Evolution under Domestication

Supplementary Figures and Tables

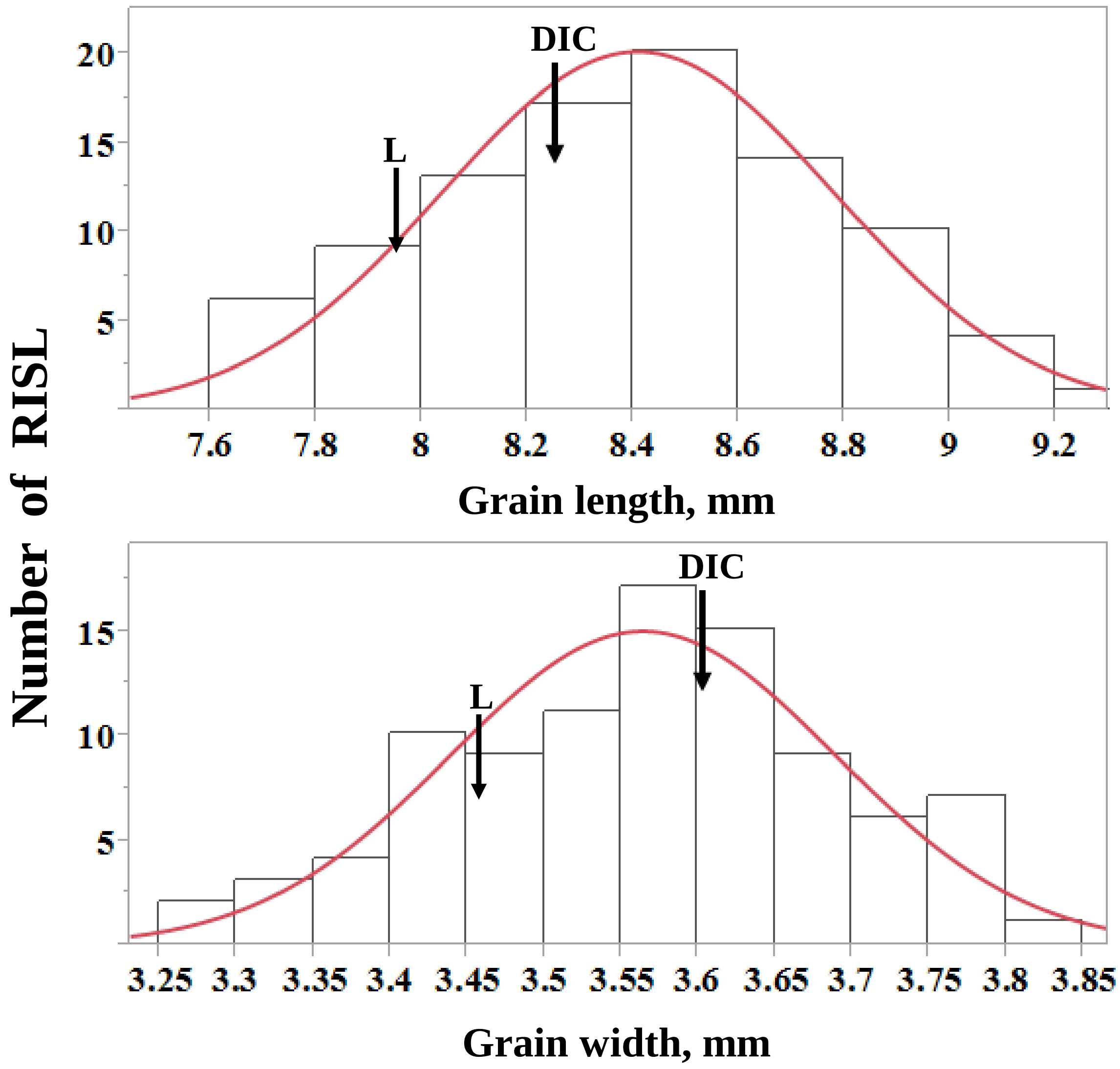

Supplementary Figure S1. Frequency distribution of grain shape parameters among the 94 RISLs. Arrows indicate the mean value for the two parental lines: Langdon (L) and DIC-2A (DIC).

Supplementary Table S1. List of wild emmer accessions and durum cultivars used in this study.

	Species	Genotype	Origin
**Wild emmer accessions**			
	T. turgidum ssp. *dicoccoides*	J28	Jordan
	T. turgidum ssp. *dicoccoides*	12-3	Israel
	T. turgidum ssp. *dicoccoides*	28-6	Israel
	T. turgidum ssp. *dicoccoides*	13-B-53	Israel
	T. turgidum ssp. *dicoccoides*	16-34	Israel
	T. turgidum ssp. *dicoccoides*	24-39	Israel
	T. turgidum ssp. *dicoccoides*	dic47	Israel
	T. turgidum ssp. *dicoccoides*	dic52	Israel
	T. turgidum ssp. *dicoccoides*	MM 1/1	Israel
	T. turgidum ssp. *dicoccoides*	MM 5/3	Israel
	T. turgidum ssp. *dicoccoides*	dic110	Turkey
	T. turgidum ssp. *dicoccoides*	dic55	Turkey
**Durum cultivars**			
	T. turgidum ssp. *durum*	C-43	Israel
	T. turgidum ssp. *durum*	C-61	Israel
	T. turgidum ssp.*durum*	C-9	Israel
	T. turgidum ssp. *durum*	P9	Israel
	T. turgidum ssp. *durum*	Eliav	Israel
	T. turgidum ssp. *durum*	Givati	Israel
	T. turgidum ssp. *durum*	Inbar	Israel
	T. turgidum ssp. *durum*	Svevo	Italy
	T. turgidum ssp. *durum*	Uzan	Israel
	T. turgidum ssp. *durum*	Kizilitan	Turkey
	T. turgidum ssp. *durum*	Kofa	USA
	T. turgidum ssp. *durum*	UC 1113	USA

Supplementary Table S2. List of primers that were developed for the current study.

Primer	Primer sequence	Corresponding GenBank accession	Polymorphism	Amplicon length
Xhuj001_F	AGCGTCTTAGTACCCTGCTTG	AK331946.1	Indel	177
Xhuj001_R	CGTGTTGGCCATGCATAAAAC			
Xhuj002_F	CTACCAAATGTGATGCCCGG	AK335551.1	Indel	177
Xhuj002_R	GGGAAGTCAAGCGTGTCTGA			
Xhuj003_F	CCTCCTGACTCCTCCCTAAA	BE497494.1	SNP	324
Xhuj003_R	TGGTAAACCAAAGGTGATAACG			
Xhuj004_F	CCGAGTTTACCCCATACCAAT	M94726.1	Indel	1066
Xhuj004_R	TGGTAAGATTTTGTTATCGCATTT			

Supplementary Table S3. Correlation values (r) among grain and embryo traits measured in RISL population.

	Grain weight	Grain length	Grain width
Grain length	0.72****		
Grain width	0.83****	0.68****	
Embryo weight	0.56****	0.33**	0.55****

** and **** indicate significant correlation between traits at P <0.01 and P<0.0001, respectively.

Supplementary Table S4. Biometric parameters of QTLs affecting grain and embryo parameters in RISL population.

Trait	Position (cM)	Nearest marker	LOD[a]	P.E.V%[b]	d[c]	Favorable allele[d]
**Grain weight**	57.8±3.15	*Xhbg494*	12.81***	0.481	5.741±0.654	Dic
**Embryo weight**	47.97±5.21	*Xbarc201*	7.7***	0.318	0.06±0.009	Dic
**Grain length**	62.38±0.97	*Xcfa2043*	12.45***	0.456	0.498±0.056	Dic
**Grain width**	56.40±4.65	*Xwmc794*	9.75***	0.382	0.154±0.02	Dic

*** indicate significance at *P* <0.001..

a LOD scores that were found to be significant when comparing hypotheses H_1 (there is a QTL in the chromosome) and H_0 (no effect of the chromosome on the trait), using the 1000 permutation test (Churchill and Doerge, 1994).

b Proportion of explained variance of the trait.

c The additive effect of an allele calculated as one-half of the mean difference between homozygotes with and without the allele.

d Favorable parental allele contributing to higher values; Langdon (L) and DIC-2A (DIC).

Supplementary Table S5. Dry weight of selected RISL seedlings with different grain-weight (GW) and embryo-weight (EmW) alleles of domesticated (LDN) and wild emmer (DIC) wheat.

RISL	Grain	Embryo	Mean±SD (mg)
**LDN**	LDN	LDN	77.75±1.92
**59**	LDN	LDN	86.62±5.48
**45**	LDN	DIC	106.27±6.21
**69**	LDN	DIC	96.49±9.36
**84**	LDN	DIC	94.03±9.70
**58**	DIC	LDN	85.00±15.51
**64**	DIC	LDN	74.54±10.35
**85**	DIC	LDN	74.17±7.77
**1**	DIC	DIC	98.45±7.70
**90**	DIC	DIC	98.45±11.35
**DIC-2A**	DIC	DIC	86.98±9.34
